# *Hemidesmus indicus* induces immunogenic death in human colorectal cancer cells

**DOI:** 10.18632/oncotarget.25325

**Published:** 2018-05-11

**Authors:** Eleonora Turrini, Elena Catanzaro, Manuele G. Muraro, Valeria Governa, Emanuele Trella, Valentina Mele, Cinzia Calcabrini, Fabiana Morroni, Giulia Sita, Patrizia Hrelia, Massimo Tacchini, Carmela Fimognari

**Affiliations:** ^1^ Department for Life Quality Studies, Alma Mater Studiorum–University of Bologna, Rimini, Italy; ^2^ Oncology Surgery, Department of Biomedicine, University Hospital of Basel and University of Basel, ZLF, Basel-Switzerland; ^3^ Cancer Immunotherapy, Department of Biomedicine, University Hospital of Basel and University of Basel, ZLF, Basel-Switzerland; ^4^ Department of Pharmacy and Biotechnology, Alma Mater Studiorum–University of Bologna, Bologna, Italy; ^5^ Department of Life Sciences and Biotechnology, University of Ferrara, Ferrara, Italy

**Keywords:** Hemidesmus indicus, immunogenic cell death, adjuvant activity, botanical drugs, colorectal cancer

## Abstract

The ability of anticancer treatments to promote the activation of tumor-reactive adaptive immune responses is emerging as a critical requirement underlying their clinical effectiveness. We investigated the ability of *Hemidesmus indicus*, a promising anticancer botanical drug, to stimulate immunogenic cell death in a human colorectal cancer cell line (DLD1). Here we show that *Hemidesmus* treatment induces tumor cell cytotoxicity characterized by surface expression of calreticulin, increased HSP70 expression and release of ATP and HMGB1. Remarkably, the exposure to released ICD-inducer factors from *Hemidesmus*-treated DLD1 cells caused a modest induction of CD14-derived dendritic cells maturation, as demonstrated by the increased expression of CD83. Moreover, at sub-toxic concentrations, H.i. treatment of monocytes and dendritic cells induced their mild activation, suggesting its additional direct immunostimulatory activity. These data indicate that *Hemidesmus indicus* induces immunogenic cell death in human tumor cells and suggest its potential relevance in innovative cancer immunotherapy protocols.

## INTRODUCTION

According to the FDA guidelines, a botanical drug is set up from a botanical drug substance and is proposed for use as a drug. In this record, FDA, without precedent for its history, proposes to endorse botanical drugs in extract as a new class of drugs. A standard FDA-approved drug is constituted by a well-characterized active principle. Conversely, a botanical drug, by definition, is made out of multiple compounds [1]. Such complex composition may give advantages, particularly in managing complex diseases with polymorphic nature that cannot respond to the standard single drugs.

Some natural products have been found to stimulate antitumor immune response [2]. For instance, an extract from the Japanese traditional medicine *Juzen-taho-to* composed of 10 medicinal plants prompted a CD8 T-cell-immunity-based anticancer response in a murine melanoma model [3]. A gallotannin-rich standardized fraction from *Caesalpinia spinosa* is endowed with immune system dependent-anticancer activity. Indeed, it induces immunogenic cell death (ICD), dendritic cells (DCs) activation, and increased generation of melanoma associated antigen-specific T cells [4].

A number of studies indicate that responsiveness to specific anticancer drugs is critically dependent on the host immune system [5, 6]. Indeed, defined cytotoxic or genotoxic agents promote the generation of anticancer immune responses, potentially leading to tumor eradication, by inducing, in malignant cells, ICD, which has emerged as a cornerstone in anticancer therapy [6]. ICD may indeed favor the generation of tumor-specific T-cell responses by recruiting and activating antigen presenting cells (APC) [7]. ICD is typically characterized by expression and/or release of damage-associated molecular patterns (DAMPs) including ATP, a “find me” signal for monocytes, and high mobility group box 1 (HMGB1), promoting the presentation of tumor-associated antigens by DCs. DAMPs also include “eat me” signals represented by exposure of calreticulin (CRT) and heat shock proteins (HSP) 70 and 90 on dying cancer cell membranes [8]. This evidence suggests that the elucidation of malignant cell death modalities is a fundamental step in the characterization of the effects of innovative antitumor strategies.

Although botanical drugs can be useful in facing complex pathologies, there are many challenges for developing botanical drugs with a well demonstrated batch-to-batch quality consistency [4]. Recently, we obtained a decoction from plant roots of *Hemidesmus indicus* (H.i.), an Indian weed widely investigated for its pharmacological properties both *in vitro* and *in vivo* [9]. Based on the results of a phytochemical screening performed on three different batches, the decoction contains 2-hydroxy-4-methoxybenzaldehyde, 3-hydroxy-4-methoxybenzaldehyde and 2-hydroxy-4-methoxybenzoic acid. Of note, the difference among the batches in the phytomarker content and pharmacological activity resulted not significant [10, 11]. In a previous study, we showed that the decoction has antitumor activity in different leukemic cell lines and enhances the antitumor activity of different antitumor drugs. Moreover, a clinically relevant observation is its ability to exhibit a cytotoxic activity in hypoxia and on blasts from recidivant patients, two conditions associated with pharmacoresistance [10, 11]. Based on those interesting pharmacological activities, here we investigate the ability of H.i. to induce ICD.

In this study, we show that treatment with H.i. induces ICD in a colorectal cancer (CRC) cell line. We further demonstrate that H.i. stimulates an enhancement of CD83 and costimulatory molecules expression on DCs cells.

## RESULTS AND DISCUSSION

### Effects of H.i. on tumor cell viability: cytotoxicity and apoptosis

**Figure 1:**
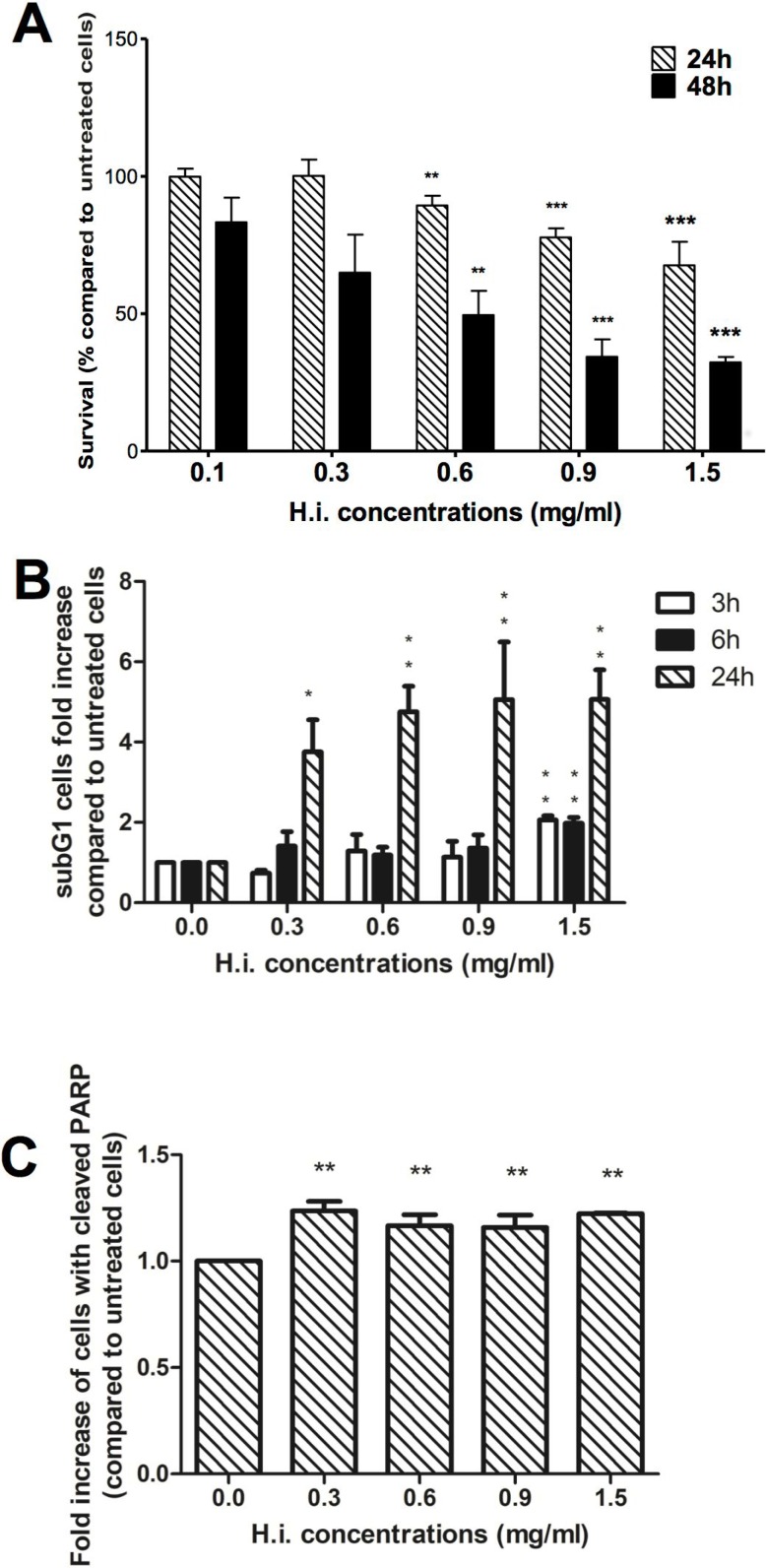
H.i. induces apoptosis on DLD1 cells Cytotoxicity of DLD1 after 24 and 48 h from H.i. treatment (0.1–1.5 mg/ml). The histograms show the decrease in DLD1 viability recorded with APH test (**A**). Sub-G1 cell population after 3, 6 and 24 h (**B**) and cleaved PARP expression after 24 h from H.i. treatment (0.3–1.5 mg/ml) (**C**). Data are the mean of at least three different experiments. ^*^p < 0.05; ^**^p < 0.01; ^***^p < 0.001.

The study begun with the investigation of H.i. concentrations needed to demise more than half of the tumor population. We treated mismatch repair (MMR)-deficient DLD1 CRC cells with increasing concentrations of H.i. for 24 and 48 h. After 48 h, 0.6, 0.9, and 1.5 mg/ml caused 52.7, 62.1 and 68.8% loss of cell viability, respectively, compared to untreated cells (Figure 1A). Those concentrations were thus selected to perform the ICD studies, while non-cytotoxic concentrations were selected to assess its immunomodulatory effect and determine its profile as adjuvant.

As ICD is a particular form of apoptotic cell death, we investigated the cell death modalities that were engaged in response to H.i. To evaluate the ability of H.i. to induce apoptosis on DLD1 cells, we analyzed the entity of cells with fractional DNA content from permeabilized cells, the so-called “sub-G1 population”. After 3 h, only H.i. 1.5 mg/ml caused a significant increase in the sub-G1 population compared to untreated cells (2-fold increase), while after 24 h treatment all tested concentrations induced a rise of sub-G1 cells with a ceiling of 5 times more than untreated cells for both 0.9 and 1.5 mg/ml (Figure 1B). To confirm that the observed sub-G1 peak was due to apoptosis, we analyzed the expression of 85 kDa fragment of cleaved poly ADP-ribose polymerase (PARP), an important reporter for caspase 3 activation. The expression of cleaved PARP analyzed after 24 h treatment was significantly higher, compared to untreated cells, at all tested concentrations (Figure 1C).

### H.i. induces endoplasmic reticulum (ER) and oxidative stress

Doxorubicin, mitoxantrone, oxaliplatin and the hypericin-based photodynamic therapy (Hyp-PDT) are all examples of ICD promoters. Nevertheless, it's hard to find similarities among them in their precise mechanism of action or molecular structure, but apparently they all induce ICD acting through a different interpretation of the same pathway. To this respect, without a sharp structure–activity relationship, the idea of employing a decoction to induce ICD, benefitting from a mix of active compounds that act jointly in a multi-target way, could overpass the challenge to identify a single molecule. What all ICD inducers share is the ability to trigger ER and oxidative stress in a concerted way, events that are officially recognized as the engine that drives the immunogenicity of ICD [12]. Thus, ascertained that H.i. induces apoptosis, we evaluated its ability to trigger those two crucial events required to convert tolerogenic apoptosis to immunogenic.

**Figure 2:**
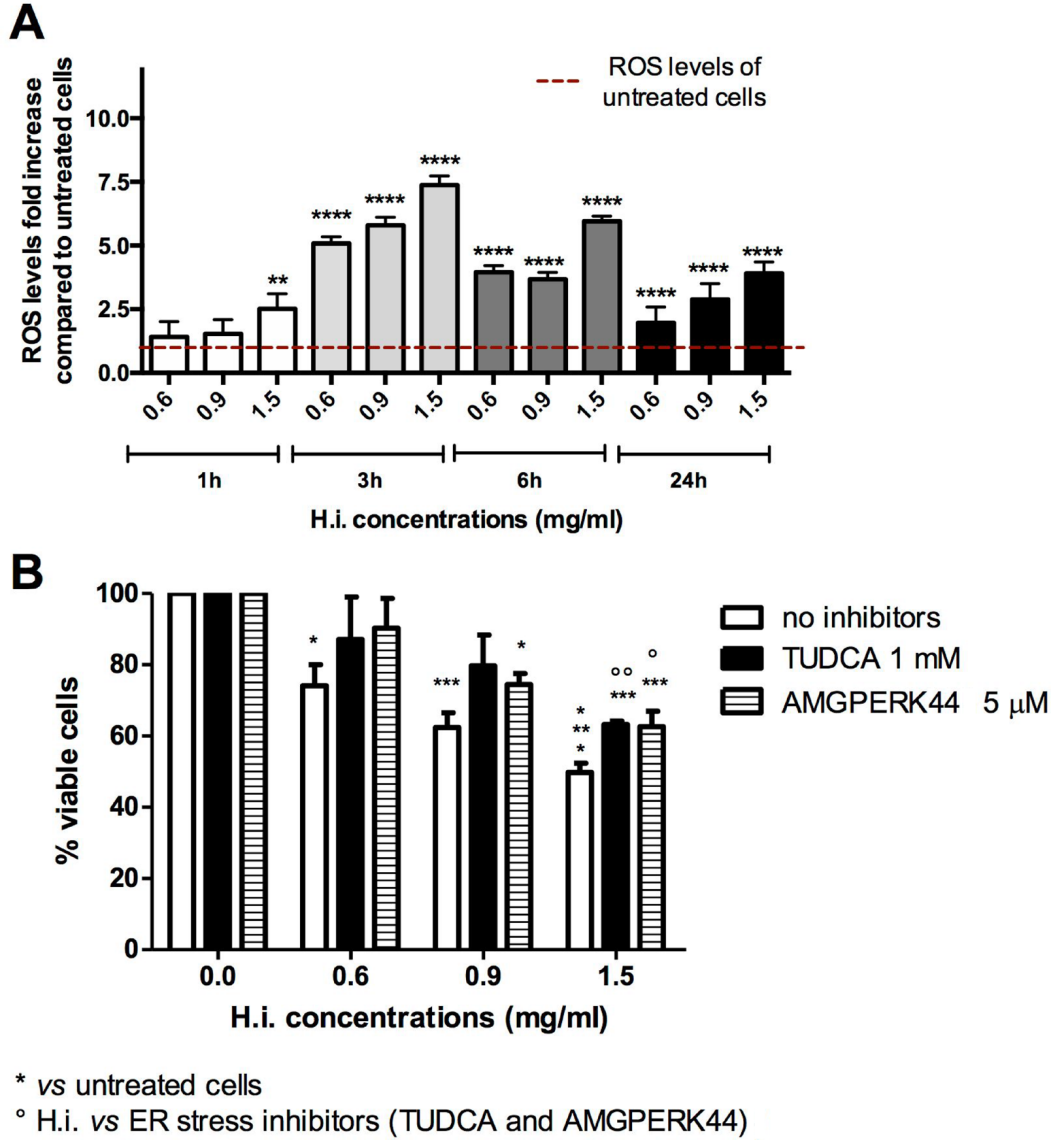
H.i. induces ER and oxidative stress Intracellular ROS fold-increase in DLD1 cells after 1, 3, 6 and 24 h from H.i. treatment at increasing concentrations (0.6–1.5 mg/ml) compared to untreated cells (**A**). Percentage of viable cells after DLD1 treatment with H.i. alone or in combination with TUDCA or AMGPERK44 for 24 h (**B**). Data are the mean of at least three different experiments. ^*^p < 0.05; ^**^p < 0.01; ^***^p < 0.001; ^****^p < 0.0001.

First, H.i.'s ability to provoke oxidative stress was examined. Intracellular ROS levels were recorded after 1, 3, 6 or 24 h of treatment. Oxidative stress was increased starting from 1 h in a concentration-dependent manner. After 3 h at all tested concentrations, H.i. led to the highest increase of ROS, as indicated by the increased DCF fluorescence: 5.08-, 5.79-, and 7.38-fold increase at 0.6, 0.9 and 1.5 mg/ml, respectively, compared to untreated cells (Figure 2A). At 6 and 24 h, the ROS levels were still concentration-dependent and above those recorded for untreated cells, but a downward trend was observed (Figure 2A).

Beside ROS production, the other pivotal event eliciting ICD is ER stress. To directly link ER stress to the antitumor potential of H.i., that event was prevented with specific agents before treating DLD1 with H.i., and then cell viability recorded. Attenuation of ER stress with both tauroursodeoxycholic acid (TUDCA, a general ER stress inhibitor) and AMGPERK44 (PERK-activation inhibitor) reduced the cytotoxic activity of H.i. observed after 24 h in a concentration-dependent manner, which however was not enough to restore complete viability (Figure 2B). This suggests a crucial, but not exhaustive, role of ER stress in H.i. mechanism of action. These results are not surprising since the decoction of H.i. is a mix of several and possibly active molecules. Those phytochemicals can weakly interact with different molecular targets and partially block different nodes within the cancer cellular network. This partial inhibition could be enough to lead to a specific antitumor effect [13]. It's interesting to underline that the cytoprotective effect of TUDCA did not differ from that of AMGPERK44, suggesting the complete involvement of PERK branch of the unfolded protein response (UPR) in the ER stress induced by H.i. This evidence is very intriguing since it has been demonstrated that, while all UPR sensors are activated during ICD, only the activation of PERK is mandatory to elicit immunogenicity [14].

Taken together, these data demonstrate that oxidative and ER stress is involved in H.i.-mediated mechanism of action and represent a perfect foundation for the following ICD studies.

### H.i. induces DAMPs trafficking

The immunogenic characteristics of ICD are due to the mobilization of DAMPs. DAMPs are endogenous molecules that acquire immunostimulant properties when exposed on the outer cellular membrane or released in the extracellular matrix, in a defined spatiotemporal manner that differs on which agent/*stimulus* started the process. When exposed or liberated into the cellular matrix by damaged or dying cells, DAMPs bind pattern recognition receptors (PRRs) directly on the surface of immune cells, such as different types of toll-like receptors (TLRs), creating a bond between the demising cancer cells and the immune system [14]. In other words, they turn into “eat me” or “find me” signals for APC, like DCs, macrophages, certain T-cells, or natural killer (NK) cells. Moreover, they foster DCs maturation and the activation, elaboration and presentation of the tumor-associated antigen (TAA) to immature T lymphocytes. In this way, the host immune system establishes the first contact with the specific TAA, and a subsequent stimulation of TAA-specific T-cell-mediated immune responses would result into the eradication of the remaining cancer cells together with an immunological memory [8].

Hence, reached this point, we investigated whether H.i.-mediated ER and oxidative stress was sufficient to trigger the particular pathway needed to reach the immunogenicity. Thus, we proceeded with the analysis of the sequential mobilization of the main *in vitro* hallmarks of ICD *i.e.* the most characterized DAMPs.

Each single DAMP has a different role in the induction of immunogenicity. Both the entity and the kinetic of DAMPs mobilization are crucial to define the immunogenicity of the demising cells. Even if ICD inducers do not share fixed patterns in terms of DAMP's trafficking (or common pathways have not been discovered yet), a sort of spatiotemporal code through which DAMPs exert their immunological awakening has now been acknowledged [8, 15]. CRT is the first DAMP exposed when ICD is triggered. This event starts before the apoptotic machine is activated since it actually precedes the externalization of phosphatidylserine [16]. Ecto-CRT has an “eat me signal” function that promotes the stimulation of phagocytic signals on APC, such as DCs and macrophages. As far as it is known, CRT exposure represents the limiting factor for ICD induction. From anthracyclines to mitoxantrone, from oxaliplatin to bortezomib, from Hyp-PDT to the oncolytic virus therapy, all *bona fide* ICD inducers require ecto-CRT in the early stages of cell death to prompt the immune system [15, 17, 18]. For instance, the only difference between the ICD inducer oxaliplatin and the immunogenic-null cisplatin is the ability to promote CRT mobilization. Indeed, if an ER stressor able to promote CRT mobilization is coupled with cisplatin, the immunogenicity establishes. At the same time, if CRT trafficking is blocked, oxaliplatin fails to induce the anticancer immune response [19, 20]. More, mitoxantrone-mediated ICD on mouse colon cancer cells (CT26) failed if CRT was silenced [17, 18]. Right after CRT exposure, other early “eat me signals” are represented by ecto-HSP70 and 90. They interact with TLRs on DCs and NK cells, fostering their maturation [21–24]. All types of stressed cells, such as necrotic or autophagic, secrete ATP, and during ICD it happens too. Even if this event does not belong to ICD only, it has been shown as a mandatory event to preserve immunogenicity: limiting ATP release translates into a diminished recruitment of immune effector cells. ATP acts as a “find me” signal attracting DCs and other myeloid cells and stimulating the proteolytic maturation of IL-1β [25, 26]. HMGB1, in turn, is released when the nucleus is damaged and is one of the last DAMPs to be emitted. It has both pro-inflammatory and DC promoting antigen presentation activity [27–30]. To name a few, mitoxantrone and oxaliplatin prompt pre-apoptotic ecto-CRT, early apoptotic secreted ATP, mid to late ecto-HSP70, and late apoptotic passive release of HMGB1 [22, 31, 32]. Shikonin, for its part, induces early to mid apoptotic ecto-CRT and ecto-HSP70 [33, 34], while Hyp-PDT causes pre-apoptotic induction of ecto-CRT and actives pre-apoptotic secretion of ATP in stressed cells, overlapping PERK-orchestrated pathways. These events are tailed by late apoptotic mobilization of HSP70 and HSP90 [21].

H.i. was tested in a time-course experimental setting for its ability to endorse surface exposure of CRT, HSP70 and 90 on non-permeabilized cells and the release in the culture medium of ATP and HMGB1.

**Figure 3:**
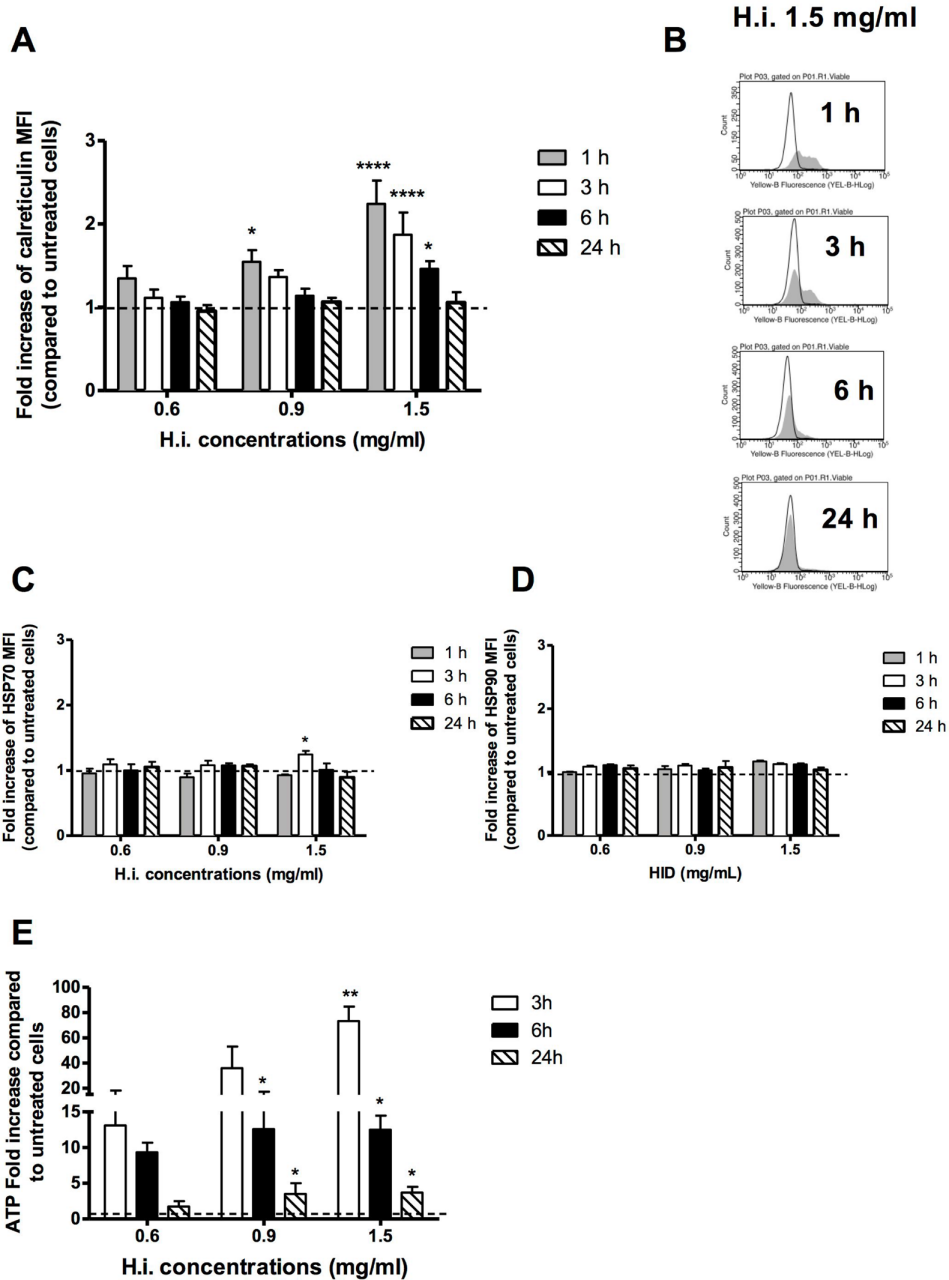
H.i. induces DAMPs trafficking Ecto-CRT (**A**, **B**), ecto-HSP70 (**C**), HSP90 (**D**) in non-permeabilized cells after 1, 3, 6, 24 h from H.i. treatment at increasing concentrations (0.6–1.5 mg/ml) and ATP secretion (**E**) after 3, 6 and 24 h from H.i. treatment. Data are the mean of at least three different experiments. ^*^p < 0.05; ^**^p < 0.01; ^****^p < 0.0001.

All tested concentrations increased the expression of ecto-CRT from 1 h treatment in a concentration-dependent manner, showing a pre-apoptotic mobilization (apoptosis was detected for the first time after 3 h treatments). Accordingly, the higher ecto-CRT values were reached after 1 h for all tested concentrations and decreased time-dependently until reaching the physiologic values after 24 h. For instance, 1.5 mg/ml induced a significant exposure of CRT with a fold increase of 2.24, 1.87 and 1.46, respectively, for 1, 3 and 6 h treatments, compared to untreated cells (Figure 3A, 3B). The amount of ecto-CRT mobilized by H.i. was very similar to that observed for Hyp-PDT, the most effective *bona fide* ICD inducer renown so far, in T24 human bladder carcinoma cells after 0.5 h of treatment [21]. Time-wise, right after CRT, we recorded HSP70 engagement. The lowest tested concentrations of H.i. did not modulate HSP70 trafficking at any time point, but 1.5 mg/ml favored its mobilization after 3 h of treatment, inducing a significant increase in HSP70 mean fluorescence intensity (MFI), which resulted 1.24 times higher than that recorded for untreated cells (Figure 3C). No significant HSP90 externalization was recorded at any concentration and time point analyzed (Figure 3D). However, even if the more DAMPs are triggered, the more immunogenicity is expected, HSP90's activity partially overlaps the one of HSP70 and alone it does not represent the *conditio sine qua non* ICD is triggered [35]. For instance, neither doxorubicin, nor mitoxantrone, nor shikonin require HSP90 mobilization to trigger ICD [8, 15, 36]. H.i. provoked ATP secretion at all tested concentrations and time points analyzed (3, 6 and 24 h), suggesting an early apoptotic release. After 3 h, it induced the highest rise, increasing ATP levels in the extracellular medium of 36 and 73 times at 0.9 and 1.5 mg/ml, respectively (Figure 3E). The entity of this increase reminds the one induced by Hyp-PDT in T24 cells at 1 h post-treatment [21]. Moving on, after 24 h of H.i. treatment, HMGB1 was mobilized from the nucleus to the cytoplasm (data not shown), event that precedes its release from the cell in a subsequent moment [19, 22].

Taken together, our data perfectly describe the profile of DAMPs trafficking triggered by an ICD inducer. They indicate that H.i. provokes pre- and early-apoptotic exposure of CRT, early- and mid-apoptotic exposure of ATP and HSP70, and a subsequent release of HMGB1 at the late stages of cell death.

Up to here, we demonstrated that the decoction is able to concurrently endorse several DAMPs, including the most characterizing CRT and ATP, and in the recognized spatiotemporal fashion. More, conversely from anthracyclines, H.i. stimulates all DAMPs at the same concentration (1.5 mg/ml), thereby incorporating the rise of these critical immunogenic signals within a single therapeutic set-up, and indicating an easier prospective of potential clinical use [21].

Taken together, these results show a clear activation of the ICD pathway and open the way to further studies aimed at defining the immunogenic potential of the decoction.

### H.i. promotes maturation of DCs toward APC phenotype

**Figure 4:**
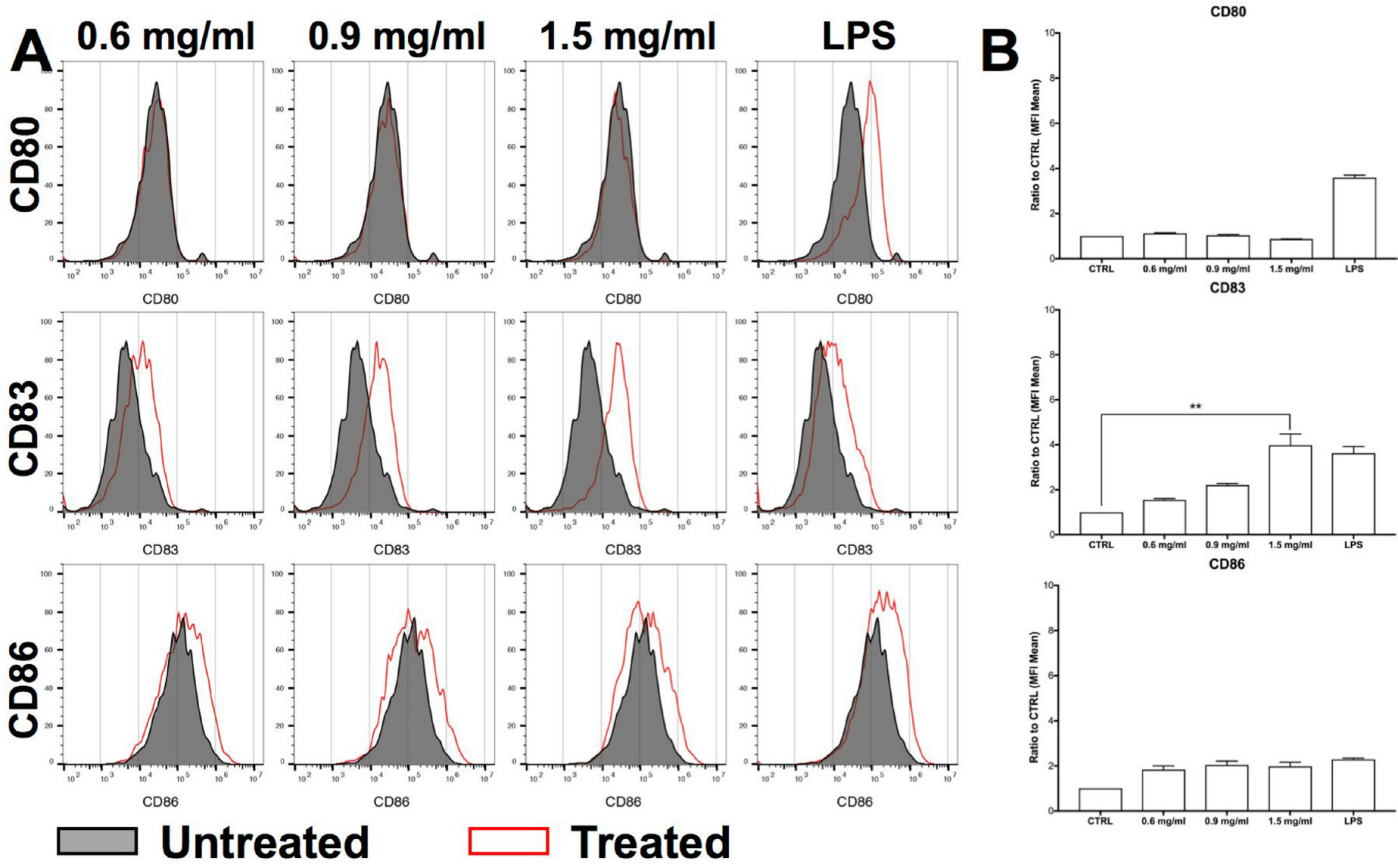
H.i. promotes iDCs maturation CD80, CD83 and CD86 expression in iDCs co-cultured with DLD1 cells (ratio 4:1) after 24 h from H.i. treatment (0.6–1.5 mg/ml) of a representative donor (**A**) and histograms representing the mean of three representative healthy donors (**B**). LPS was used as positive control. ^**^p < 0.01.

To confirm the ability of H.i. to induce ICD, we exposed CD14-derived immature DCs (iDCs) to H.i.-treated DLD1 in co-culture for 24 h. The exposure to released ICD-inducer factors from H.i.-treated DLD1 showed a modest induction of differentiation of the CD14-derived iDC. Indeed, the treated direct co-culture induced a concentration-dependent increase in the expression of CD83 (2 and 4 times higher compared to control at 0.9 mg/ml and 1.5 mg/ml, respectively), and at all doses tested the expression of CD86 was always 2 times higher than untreated co-cultures. No changes were observed in the expression of CD80 (Figure 4A and 4B). Considering that CD83 alone is a marker of DC maturation while CD80 and 86 are co-stimulatory molecules that can or cannot be expressed on mature DCs [37], our data demonstrate that H.i.-mediated cell death is able to promote the transition from an immature to a mature state of DCs. This evidence closes in on the assessment of H.i. ability to elicit all the hallmarks of ICD on human colon cancer cells. Moreover, it can provide a rationale for the design of *in vivo* experiments to investigate the “endogenous vaccine” power of H.i.-treated tumor cells.

### H.i. enhances the expression of co-stimolatory molecules on monocytes and DC cells at sub-toxic concentrations

In order to assess the immunomodulatory capacity of H.i., we first evaluated its toxic effect on total peripheral blood mononuclear cells (PBMCs) from healthy donors. We observed that at 0.6 mg/ml (dose that causes around 50% cell death at 48 h in DLD1 cells) there was no increase in the percentage of apoptotic or necrotic cells. The highest concentration (1.5 mg/ml) determined an increase in necrotic cells compared to the untreated control and to the 0.6 mg/ml dose (Supplementary Figure 2).

**Figure 5:**
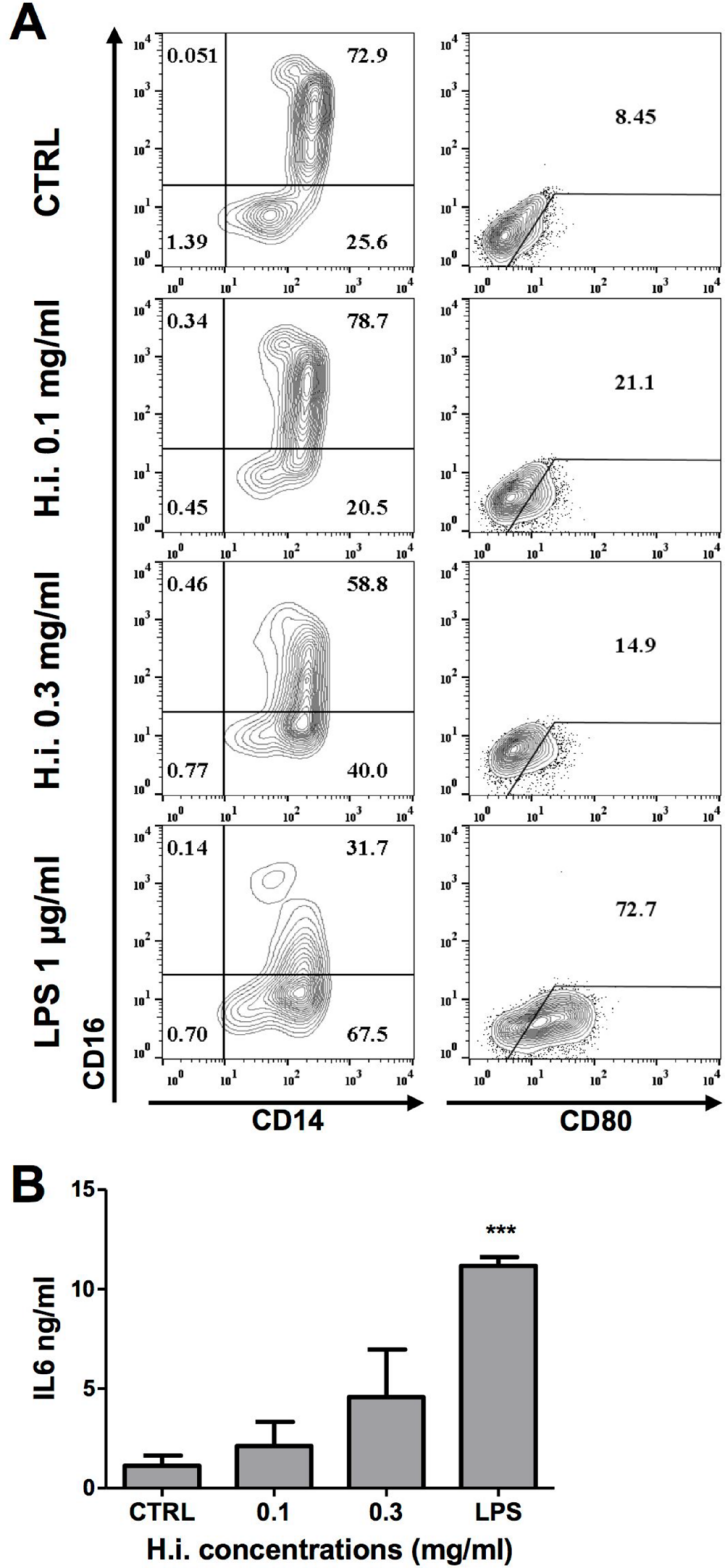
H.i. impacts on CD14 monocytes activation (**A**) CD14, CD16 and CD80 expression in monocytes from a representative donor after exposure to H.i. and LPS, as positive control, as compared to untreated cells (24 h) (A). IL6 release following treatment with H.i. of monocytes from three different healthy donors (**B**). ^***^p < 0.001.

To directly address its immunomodulatory potential, we investigated the effects of H.i. on monocytes (CD14+ cells) from healthy donors, precursors of CD14-derived iDCs. Overnight treatment with increasing concentrations of H.i. induced the down-regulation of CD16 and the upregulation of CD80 expression, although to lesser extents as compared to the positive control lipopolysaccharide (LPS) (Figure 5A). A modest increase in IL6 release was also observed as compared to untreated cells (Figure 5B).

**Figure 6:**
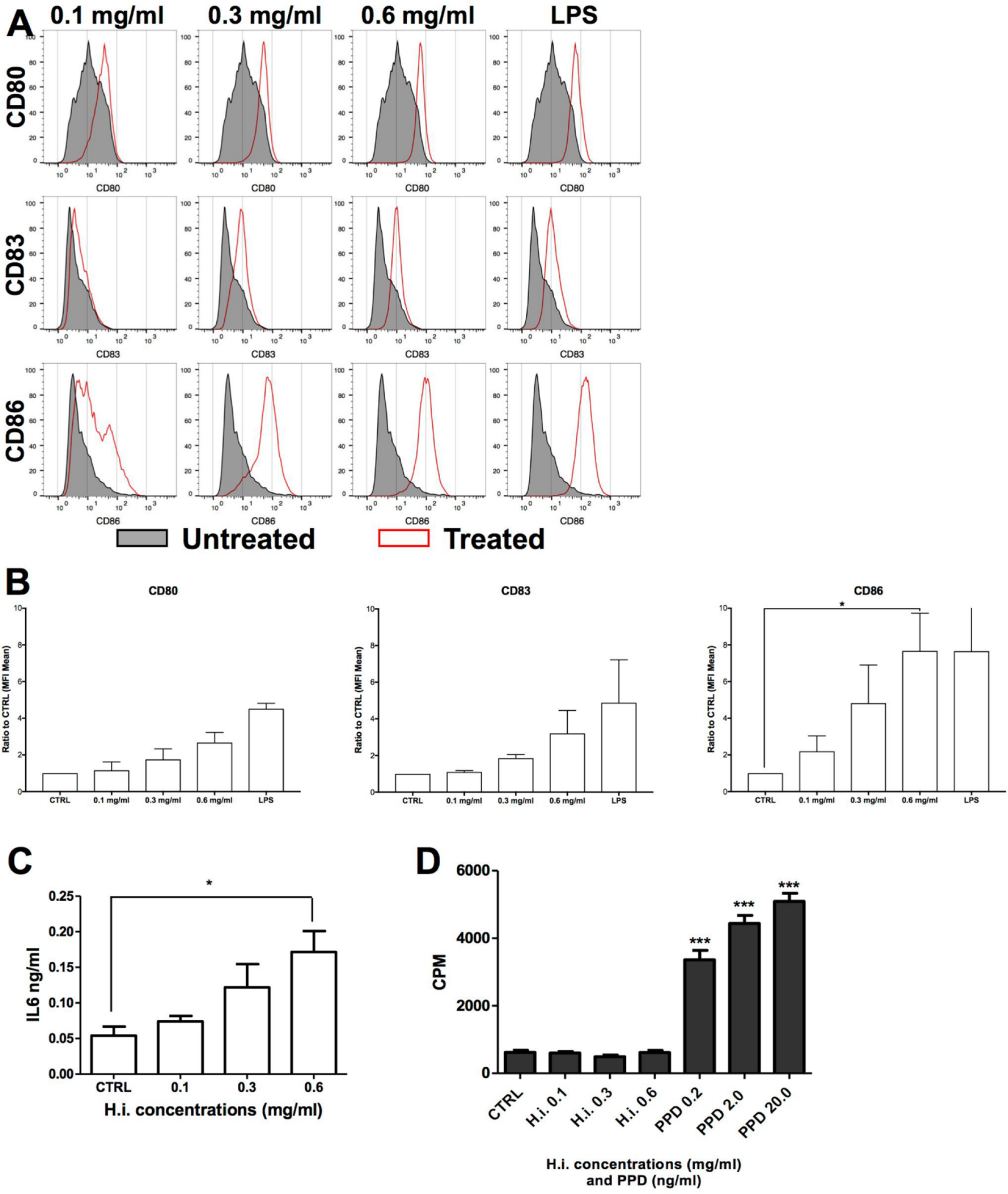
H.i. enhances the expression of CD83 and co-stimulatory molecules on iDCs CD80, CD86 and CD83 expression in iDCs untreated or following treatment with H.i. 0.1–0.6 mg/ml of a representative donor (**A**) and histograms of the mean of three donors (**B**). IL6 release following iDCs treatment with H.i. from three different healthy donors (**C**). LPS-treated cells were used as positive control. Cell proliferation (CPM) induced by H.i. (0.1–0.6 mg/ml) or tubercolin (PPD) as assessed by ^3^H-thymidine incorporation (**D**). ^*^p < 0.05; ^***^p < 0.001.

Next, we treated iDCs with H.i. for 24 h. iDC treatment with 0.1 up to 0.6 mg/ml H.i. resulted in an increased expression of CD80, CD83, and CD86 already at low doses (0.3 mg/ml). In particular, we observed that CD86 was increased more than 2-fold already at the lowest tested concentration (0.1 mg/ml), with a 7-fold increase when the DCs were treated with 0.6 mg/ml. An increase of 1.85-fold and more than 3-fold was observed for CD83 expression, respectively at 0.3 and 0.6 mg/ml. CD80 was increased 1.75-fold and more than 2-fold at 0.3 and 0.6 mg/ml concentration, respectively (Figure 6A and 6B). H.i. treatment induced also an almost 2-fold increase in IL6 release as compared to untreated cells, already at the lowest dose tested (Figure 6C). H.i. showed the potential to induce the DC maturation already at doses where its cytotoxic effect on tumor cells was not yet effective. Therefore, H.i. has the potential to act as an adjuvant agent by enhancing an immunogenic response. Finally, the antigenic potential of H.i. to be recognized by T cells was also assessed. PBMCs from healthy donors were cultured in presence of increasing concentrations of H.i. and lymphoproliferative response was analyzed by ^3^H-thymidine incorporation on day 7. Data from 4 different experiments with cells from healthy donors indicate that H.i. did not *per se* induce T-cell proliferation (Figure 6D).

## MATERIALS AND METHODS

### H.i. decoction preparation

H.i. decoction was prepared according to the procedures described in the Ajurvedic Pharmacopeia of India. The plant (voucher #MAPL/20/178) was collected from Ram Bagh in Rajastan, India, authenticated by Dr. MR Uniyal, Maharishi Ayurveda Product Ltd (Noida, India) as described in Fimognari *et al*. 2011 [11]. Our previous HPLC analyses demonstrated and quantified the presence of three main phytomarkers (2-hydroxy-4-methoxybenzaldehyde, 3-hydroxy-4-methoxybenzaldehyde and 2-hydroxy-4-methoxybenzoic acid) in the decoction [11].

### Cell cultures

An authenticated human colon adenocarcinoma cell line (DLD1), MMR-deficient, was purchased from ATCC (Manassas, Virginia, USA). DLD1 cells were propagated in adhesion and cultured in RPMI 1640, supplemented with 10% inactivated fetal bovine serum, 1% GlutaMAX-I, and 1% kanamycin (all purchased by Thermo Fisher Scientific, Lucerne, Switzerland), at 37°C and 5% CO_2_. All cultures were tested by PCR and proven to be mycoplasma free prior to experimental investigations.

PBMCs from healthy donors (Blood donor center of the University Hospital of Basel) were obtained by gradient centrifugation. All donors provided written informed consent. CD14+ monocytes were magnetically isolated from PBMCs by using antibody-coated beads (Miltenyi Biotech, Bergisch Gladbach, Germany) and cultured in RPMI 1640 supplemented with 1% GlutaMAX-I, 1% non-essential amino acids (NEAA), 1% sodium pyruvate, HEPES, 1% kanamycin sulfate, and 10% fetal bovine serum (all purchased by Thermo Fisher Scientific) for CD14+ monocytes. DCs were generated in presence of GM-CSF and IL4, as previously described [25].

### Cell treatment

Cells were treated with H.i. decoction at the indicated concentrations. To rule out a role of contaminating endotoxins in the elicitation of the effects of H.i., polimixin B (Calbiochem, Zug, Switzerland) was added at 10 μg/ml to all cultures during cell treatment with H.i. DLD1 cells were pre-treated (1 h) and co-treated (24 h) with ER stress inhibitors such as the chemical chaperone TUDCA (Sigma Aldrich) 1 mM and AMGPERK44 (Tocris Bioscience, Bristol, United Kingdom) 5 μM.

In order to assess DC maturation or CD14 activation, CD14, iDCs, or co-culture of DLD1 cells and iDCs (4:1), were treated with H.i. decoction (Supplementary Figure 1). LPS 1 μg/ml was used as positive control.

### Cell viability

The acid phosphatase (APH) assay was used to determine cell viability [47]. After 24 and 48 h of treatment, cells were washed twice with PBS and then 100 μL PBS, 100 μl of the assay buffer [0.1 M sodium acetate, 0.1% Triton-X-100, supplemented with 4-nitrophenyl phosphate disodium salt hexahydrate (N9389, Sigma Aldrich)] were added to each well. After 90 min incubation at 37°C, 10 μl NaOH 1 N was added to each well and the absorption at 405 nm was measured within 10 min on a microplate analyzer. 405 nm absorbance is directly proportional to numbers of viable cells.

A second test was employed to record cell viability, using 4-methylumbelliferyl heptanoate (MUH, Sigma Aldrich) that becomes highly fluorescent after hydrolysis of the ester linkage and, thus, measures cellular lipase and esterase activity [48]. After cell treatment with ER stress inhibitors and H.i., cells were washed with PBS and incubated with MUH 1 mg/ml. After 30 min of incubation at 37°C and 5% CO_2_, fluorescence was measured (330 nm excitation; 450 nm emission) using the microplate reader Victor X3 (Perkin Elmer, Walthman, MA, USA).

PBMCs cell viability was assessed through annexin V/propidium iodide (PI) staining and analyzed by flow cytometry.

### Apoptosis detection

After 3, 6 and 24 h from H.i. treatment, cells were permeabilized for 30 min with ethanol 70% and then stained with the DNA intercalating dye PI to detect cells with reduced DNA content, which is represented by the sub-G1 population. To confirm that the observed fractional content of DNA was due to apoptotic cell death, we analyzed the expression of 85 kDa fragment of cleaved PARP. Briefly, after 24 h H.i. treatment, 1 × 10^6^ cells were fixed and permeabilized by 2% of paraformaldehyde in PBS 1x and 90% methanol, respectively. Cells were then incubated for 30 min with fluorescein isothiocyanate cleaved PARP antibody (1:100, Invitrogen, Carlsbad, CA, USA). Samples were analyzed via flow cytometry and the fold increase of MFI compared to untreated cells was recorded.

### ROS detection

Intracellular ROS levels after 1, 3, 6 and 24 h H.i. treatment (0.0–1.5 mg/ml) were measured through dichlorofluorescein (DCF) assay. Briefly, 20 min before the end of H.i. treatment, DLD1 cells were incubated with 10 μM 2′,7′ – dichlorodihydrofluoresceine diacetate (H_2_-DCFDA) (Sigma) at 37°C and 5% CO_2_. Cells were washed with PBS 1x, trypsinized, and measured for oxidation of H_2_-DCFDA by fluorescence microscopic analysis using Nikon Eclipse Ti equipped with Digital Sight camera DS U3 (Nikon, Tokyo, Japan). Fluorescence images were digitally acquired and processed for fluorescence intensity using the analysis software Image J. Mean fluorescence values were determined by averaging the fluorescence of at least 100 cells/treatment conditions. The experiment was performed in triplicate.

### Flow cytometry

Fluorochrome-labeled monoclonal antibodies recognizing CD1a, CD14, CD16, CD83, CD80, CD86, were obtained from Becton Dickinson (Allschwil, Switzerland); CRT, HSP70- and HSP90-specific labeled antibodies were obtained from Abcam (Cambridge, United Kingdom). After treatment with the decoction, cells were collected with TryPLE Express (Thermo Fisher Scientific) and stained for 20 min with the appropriate antibody. Specific binding was evaluated by flow cytometry [FACScalibur (Becton Dickinson); Guava EasyCyte 6-2L (Merck, Darmstadt, Germany); CytoFLEX (Beckman Coulter, Brea, CA, USA)]. PI or Sytox Red (Thermo Fisher Scientific) were added to each analyzed sample to discriminate live and dead cells. The permeabilized cells were excluded from DAMPs analysis, and the fold changes in the MFI were analyzed.

### Immunofluorescence

Cells were cultured in 8 well culture chambers slide (Falcon, Corning, Germany) and, after treatment with H.i. for 24 h, fixed with formalin 4% and incubated with mouse monoclonal anti-HMGB1 specific antibodies (Abcam), followed by incubation with goat anti-mouse Alexa Fluor 546 secondary antibody (Invitrogen). Nuclei were counterstained with 4′,6-diamidino-2-phenylindole (DAPI). Cells were examined under an Olympus BX61 fluorescence microscope (Olympus, Volketswil, Switzerland) and images were captured by digital camera and analysis software (Soft Imaging System GmbH, Münster, Germany) with a 20×, 60× or 100× magnification.

### Extracellular ATP detection

For the analysis of ATP extracellular levels, the kit ATPLite 1 step (Perkin Elmer, Waltham, Massachusetts, USA) was used. DLD1 cells were seeded and treated with increasing concentrations of H.i. in HBSS buffer or complete medium for 3, 6 or 24 h. At the end of incubation, supernatants were collected and ATP release was analyzed according to manufacturers’ instructions. The reaction of ATP with luciferase and D-luciferin is responsible for the production of light. The emitted light is proportional to the ATP concentration. After shaking for 2 min at 700 rpm using an orbital microplate shaker 711/CT+ (Asal srl, Cernusco, Italy), luminescence of the samples was measured using a microplate reader Victor X3 (Perkin Elmer).

### Proliferation assay

Proliferation of PBMCs obtained by healthy donors was evaluated by ^3^H-thymidyne incorporation. PBMCs cells were cultured for 5 days after treatment with H.i. 0.1–0.6 mg/ml. Cells were treated with 1 μCi/200 μl ^3^H-thymidine (Amerchem, Little Chalfont, UK) for 18 h and harvested on paper filters. ^3^H-thymidine uptake from PBMCs was measured in a liquid scintillation counter. PPD (Staten Serum Institute, Copenhagen, Denmark) 0.2, 2.0 and 20.0 ng/ml was used as positive control for ^3^H-thymidine incorporation.

### ELISA assay

The release of IL6 in supernatants was measured by ELISA using IL6-specific reagents (BD Biosciences, Allschwil, Switzerland), according to manufacturer's protocol.

### Statistical analysis

All experiments were performed at least in triplicates. Results were expressed as the mean ± SEM and were analyzed by Student's *t* test or two way ANOVA with 0.05 significance threshold. GraphPad Instat 6.0 statistical software (GraphPad Prism, San Diego, CA, USA) was used to perform all the analyses.

## CONCLUSIONS

A number of studies in the past decade consistently indicate that effectiveness of a variety of anti-cancer treatments, initially meant to directly inhibit tumor cell survival and proliferation, is actually based on immune system activation [38, 39]. ICD, induced by selected compounds or irradiation, results in the expression of so-called DAMPs, favoring the generation of a tumor microenvironment promoting the activation of APC, which, in turn, may exert anti-tumor effects *per se* and induce adaptive tumor specific T cell responses upon presentation of tumor-associated antigens [40].

This was the first report where the ability of a botanical drug to trigger all *in vitro* hallmarks of ICD was demonstrated. Indeed, the ability of different synthetic chemotherapeutic drugs to induce ICD has been characterized in detail [12], while there is a paucity of data regarding natural products of potential clinical relevance [33]. A variety of natural anticancer compounds have been successfully characterized [41, 42]. However, their immunogenic potential has not been analyzed in comparable detail [43]. Here we show that a H.i. decoction is able to induce an immunogenic type of cell death. Importantly, it appears to be devoid of intrinsic antigenic or mitogenic potential.

A typical pitfall frequently associated with the use of natural products is represented by their complex standardization. However, in the case of H.i., HPLC phytochemicals analysis has clearly demonstrated the presence of three specific phytomarkers, which can be conveniently used as fingerprints [11]. Alongside, toxicity seems not to be an issue related to botanical drugs in general [44] and to H.i. in particular. We recently demonstrated the anti-genotoxicity of an aqueous concentrated H.i. extract [45], while neither lethality nor conspicuous alteration in mice behavior was noticed after root powder suspension was administered to test acute toxicity on Swiss albino mice [46]. Furthermore, the LD_50_ (the amount of a compound sufficient to kill 50% of a population) measured for liver and kidney is at least 130 times higher than the concentrations used in this study [9]. Thus, our results identify H.i. as a potential adjuvant of other traditional antitumor agents. It has been already demonstrated that H.i. potentiates the antileukemia potential of 6-thioguanine, cytarabine and methotrexate [11], and interesting could be to verify whether the decoction triggers an immunological awakening in a combination therapy with those or other tolerogenic traditional anticancer agents. Else, since the induction of ICD alone is not always able to entirely subvert the immunosuppressive tumor microenvironment [14], H.i. might be useful in increasing responsiveness to other immunological anticancer therapies, such as monoclonal antibodies that target immunological checkpoints.

On the whole, the antitumor profile of H.i. that emerges from this study provides a clear rationale for the design of *in vivo* experiments to confirm the “endogenous vaccine” power of H.i.-treated tumor cells and underlines the clinical potential of this natural product.

## SUPPLEMENTARY MATERIALS FIGURES



## Abbreviations

APC: antigen presenting cells
APH: acid phosphatase
CPM: cell proliferation
CRT: calreticulin
CRC: colorectal cancer
DAMPs: damage-associated molecular patterns
DAPI: 4’,6-diamidino-2-phenylindole
DC: dendritic cell
DCF: dichlorofluorescein
ER: endoplasmic reticulum
H_2_-DCFDA: 2’,7’ – dichlorodihydrofluoresceine diacetate
H.i.: Hemidesmus indicus
HMGB1: high mobility group box 1
HSP: heat shock proteins
Hyp-PDT: hypericin-based photodynamic therapy
ICD: immunogenic cell death
iDC: immature DC
LD_50_: lethal dose 50
LPS: lipopolysaccharide
MFI: mean fluorescence intensity
MLR: mixed leukocyte reactions
MMR: mismatch repair
MUH: 4-methylumbelliferyl heptanoate
NEAA: non-essential amino acids
NK: natural killer
PARP: poly ADP-ribose polymerase
PRR: pattern recognition receptors
PBMCs: peripheral blood mononuclear cells
PI: propidium iodide
PPD: tubercolin
TAA: tumor-associated antigen
TLR: toll-like receptors
TUDCA: tauroursodeoxycholic acid
UPR: unfolded protein response
