# Supplementary material for: Hemidesmus indicus induces immunogenic death in human colorectal cancer cells
Source: Oncotarget. 2018 May 11;9(36):24443–56. doi: 10.18632/oncotarget.25325 (PMC5966270; doi:10.18632/oncotarget.25325)
Supplement: Supplementary file 1 [file oncotarget-09-24443-s001.pdf]

Hemidesmus indicus induces immunogenic death in human colorectal cancer cells

SUPPLEMENTARY MATERIALS

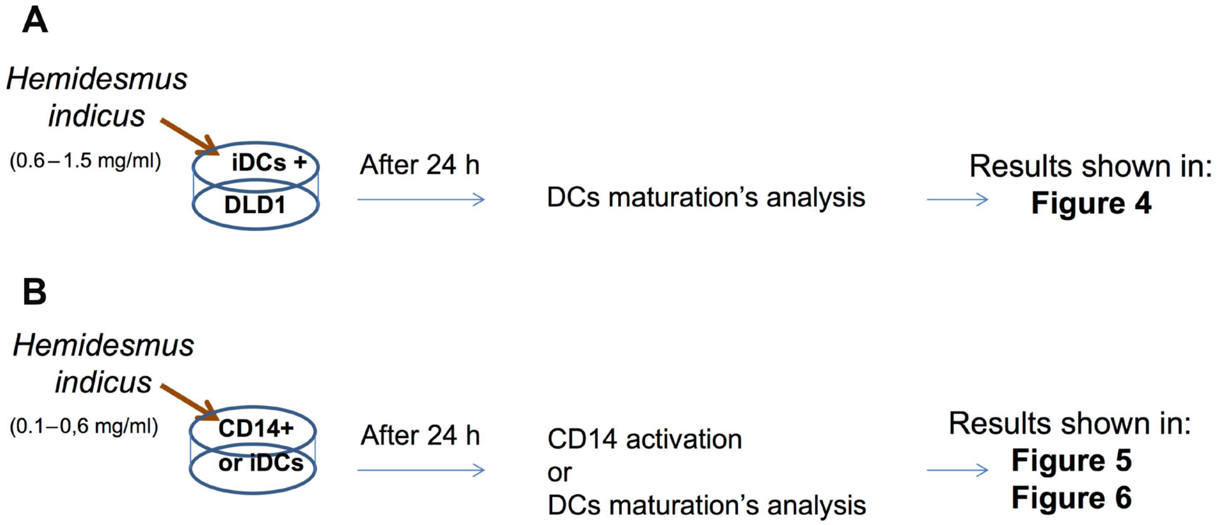

Supplementary Figure 1: Scheme of the experimental setting for H.i. treatment of monocytes and DC.

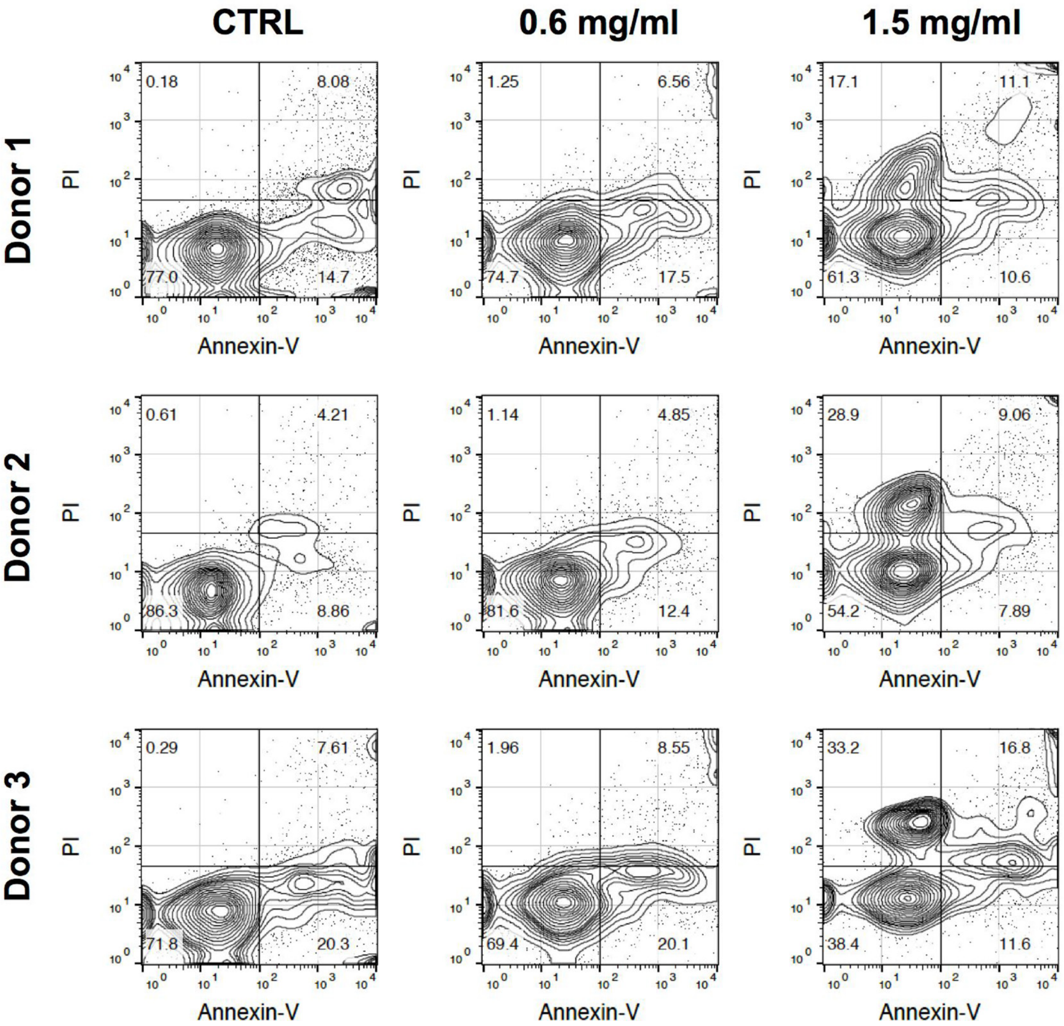

Supplementary Figure 2: PBMCs viability after H.i. treatment.
